# International Biological Reference Preparations for Epidemic Infectious Diseases

**DOI:** 10.3201/eid2502.180798

**Published:** 2019-02

**Authors:** Tommy Rampling, Mark Page, Peter Horby

**Affiliations:** Hospital for Tropical Diseases, London, UK (T. Rampling);; National Institute for Biological Standards and Control, Potter’s Bar, UK (M. Page); University of Oxford, Oxford, UK (P. Horby)

**Keywords:** biological standardization, reference preparations, infectious diseases, emerging infectious diseases, epidemic preparedness, bioterrorism and preparedness, research and development, viruses, Ebola virus disease, Middle East respiratory syndrome coronavirus, vaccines, World Health Organization, zoonoses, global health security

## Abstract

Recent years have seen unprecedented investment in research and development for countermeasures for high-threat pathogens, including specific and ambitious objectives for development of diagnostics, therapeutics, and vaccines. The inadequate availability of biological reference materials for these pathogens poses a genuine obstacle in pursuit of these objectives, and the lack of a comprehensive and equitable framework for developing reference materials is a weakness. We outline the need for internationally standardized biological materials for high-threat pathogens as a core element of global health security. We also outline the key components of a framework for addressing this deficiency.

The availability of biological reference materials, including antigens, antibodies, and nucleic acids, is essential for development of vaccines, biotherapeutics, and diagnostics (*1*). Complex macromolecules, such as immunoglobulins, or genetic material often cannot be adequately characterized by chemical or physical means alone, and reference preparations are necessary to enable consistency and comparison across assays. Immunoassays such as ELISAs or molecular methods such as quantitative PCR can be used to quantify biological materials, but these assays are subject to inherent variability between test instances and different laboratories, and require the use of reference preparations to generate a quantitative output (*2*). Furthermore, a key asset in research progress is the ability to compare results between studies and across different institutions. International reference preparations (IRPs) are internationally agreed upon reference materials that enable consistency and comparison between different studies and laboratories. Use of IRPs also ensures consistency of production and quality of biological medicinal products and is essential for establishment of appropriate clinical dosing.

## History of Biological Standardization

The concept of biological standardization and the use of biological reference materials has existed since the turn of the 20th century after simultaneous discovery of diphtheria antitoxin by von Behring and Roux (*3*,*4*). Attempts to recreate production of antitoxin in horses were successful in France, but yielded an ineffective product in England. This failure was attributed to weak serum and led Ehrlich to propose use of a standard antitoxin preparation, measured in units, with which to calibrate future batches and ensure potency (*5*). This concept was applied by Dale in the 1920s to other biological products, such as insulin, but the need for international oversight was recognized (*6*).

In the 1920s, the League of Nations initiated the provision of IRPs under the Commission on Biological Standardization, and biological standardization was subsequently incorporated into the constitution of the World Health Organization (WHO) upon its creation in 1946 (*7*). Since 1947, the provision of WHO reference materials has played a vital role in the translation of laboratory science into worldwide clinical practice and has been delivered through the WHO Expert Committee on Biological Standardization, WHO collaborating centers, and various state and nonstate partners. The Expert Committee on Biological Standardization meets annually to establish detailed recommendations and guidelines for manufacturing, licensing, and control of complex biological materials, including blood products, vaccines, and related in vitro diagnostic tests, and to maintain a catalog of IRPs to be distributed globally as required. These WHO IRPs, composed of international reference standards and other reference materials (Table 1), provide a common set of reagents that are used to ensure the quality of biological assays and medicines globally. These IRPs are considered to be the standard against which regional, national, and international laboratories and manufacturers should calibrate their own working standards. Reference materials only become WHO-endorsed IRPs after extensive collaborative studies involving multiple laboratories internationally. This process enables materials to be ascribed defined units of biological activity, most commonly international units.

**Table 1:** Types of WHO reference preparations for epidemic infectious diseases*

Types of reference material	Characteristic
International standards	Extensively characterized following international collaborative studies enable activity of biological preparations to be expressed in the same way globally, most commonly in international units
International reference reagents	Less extensively characterized than international standards. Not assigned international units. Reference reagents are interim and not intended to be replaced when they expire or are depleted.
International reference panels	Group of reference materials established to collectively aid evaluation of assays or diagnostic tests. Comply with requirements for WHO reference standards/reagents.
Working or secondary standard	Reference standards established by regional or national authorities, or by other laboratories, that are calibrated against, and traceable to, the primary WHO materials and are intended for use in routine tests.

*WHO, World Health Organization.

Although several hundred centers contribute to WHO collaborative studies, only 3 centers produce and hold IRPs. These centers are the National Institute for Biological Standards and Control in England, the Center for Biologics Evaluation and Research in the United States, and the Paul-Ehrlich Institute in Germany. The role of these institutions is to produce, store, and distribute IRPs and differs from those that distribute repository materials, such as the US Centers for Disease Control and Prevention, the International Reagent Resource, and the National Institute of Allergy and Infectious Diseases Biodefense and Emerging Infections research resources repository.

## Utility of IRPs for WHO Research and Development Blueprint Diseases

In the aftermath of the 2014 West Africa Ebola virus disease (EVD) outbreak, there has been considerable focus on expediting research and development to ensure we are better prepared for future disease outbreaks. In 2015, WHO launched the Research and Development Blueprint for Action to Prevent Epidemics, a global strategy and preparedness plan that seeks to build upon the successes and address the gaps identified during the 2014 EVD outbreak by focusing on severe, emerging diseases with the potential to create a public health emergency and for which inadequate treatment and preventive options are currently available (*8*,*9*). The blueprint is organized such that operations fall into 4 clusters of activities: 1) improving coordination, 2) accelerating research and development processes, 3) developing norms and standards, and 4) streamlining operational research and development response during outbreaks. Furthermore, the second cluster, which focused on accelerating research and development processes, is subdivided into 3 distinct areas of work: 1) assessing epidemic threats and defining priority pathogens; 2) developing research and development roadmaps to accelerate evaluation of diagnostics, therapeutics, and vaccines; and 3) outlining appropriate regulatory and ethical pathways.

Central to the research and development blueprint is a list of priority diseases, first published in November 2015, with subsequent revision through a tailored prioritization method in 2017 and 2018 (Table 2) (*10*,*11*). For each priority disease on the list, a research and development roadmap is to be produced that encompasses diagnostics, vaccines, and therapeutics. The first research and development roadmap to have been developed and published is for Middle East respiratory syndrome coronavirus (MERS-CoV) (*12*), which serves to inform the roadmap generation process for other diseases on the priority pathogen list. Within the MERS-CoV roadmap, development of reference reagents has been identified as a priority, but the major operational and ethical issues associated with sourcing and obtaining bulk starting material for such reagents have not been discussed. An allied initiative is the Coalition for Epidemic Preparedness and Innovation (CEPI), whose aim is to accelerate development of vaccines for high-threat pathogens (*13*). The necessity for reference materials in development and evaluation of new diagnostics, vaccines, and biotherapeutics has been recognized by CEPI by the formation of a Working Group on Standards and Assays, but they are not explicitly considered in the WHO research and development blueprint.

**Table 2:** Revised list of World Health Organization priority diseases, February 2018*

Disease or pathogen	Current development status of international reference preparations
Crimean-Congo hemorrhagic fever	None available. Source material required.
Ebola virus disease and Marburg virus disease	Ebola virus antibody and NA IRPs available. VP40 antigen in development. No other filovirus IRPs available. Source material required.
Lassa fever	None available. Source material required.
MERS-CoV and SARS	Antibody and NA IRPs in development for MERS-CoV. Additional source material required.
Nipah virus and related henipavirus diseases	None available. Source material required.
Rift Valley fever	None available. Source material required.
Zika	NA and antibody IRPs available.
Disease X†	Not applicable.
Diseases or pathogens considered for inclusion on priority pathogen list and under annual review	
Arenavirus hemorrhagic fevers other than Lassa fever	None available. Source material required.
Chikungunya	None available.
Highly pathogenic coronavirus diseases other than MERS-CoV and SARS	None available. Source material required.
Emergent nonpolio enteroviruses (including EV71, D68)	EV71 antibody IRP available. No other nonpolio enterovirus IRPs available. Source material required.
Severe fever with thrombocytopenia syndrome	None available. Source material required.

*EV, enterovirus; IRP, international reference preparation; MERS-CoV, Middle East respiratory syndrome coronavirus; NA, nucleic acid; SARS, severe acute respiratory syndrome; VP, virus protein. †Any disease identified before the next review by using the blueprint decision instrument will be included in the list.

Before the 2014 EVD epidemic, no IRPs existed for any diseases on the research and development bueprint. Expedited development led to production of an interim Ebola antibody reference reagent in October 2015, and the first international standard was endorsed in October 2017 (*14*,*15*). Although this accelerated production represents an impressive collaborative feat, the lack of availability of PCR, antigen, and antibody IRPs at the start of this EVD outbreak, and for subsequent outbreaks of infection with MERS-CoV and Zika virus, likely hampered development of accurate diagnostics and vaccines (*16*). In addition, the exceptional circumstances and unprecedented duration of the 2014 EVD epidemic enabled a major proportion of IRP development to occur while the outbreak was ongoing.

Development of IRPs from sample acquisition to WHO endorsement usually takes 2–3 years, which is considerably longer than most outbreaks of diseases on the research and development blueprint. The availability of IRPs for often rare and sporadic diseases with epidemic potential before, or soon after, occurrence of outbreaks would facilitate diagnostics and research activities, such as seroepidemiologic studies and immunogenicity assessment of experimental vaccines. At the present time, with the exception of *Zaire Ebolavirus* antibody, antigen, and nucleic acid IRPs and a WHO-endorsed Zika virus RNA and antibody standards, no IRPs exist for any other disease on the research and development blueprint. The availability of IRPs for these diseases would facilitate development of essential tools in epidemic preparedness, including diagnostics, vaccines, and therapeutics.

## Diagnostics

Accurate and accessible diagnostic tools are vital in limiting the public health effect of disease outbreaks, in addition to providing reliable data for clinical and epidemiologic studies. A coordinated diagnostic product development effort for epidemic disease threats has been initiated by the Foundation for Innovative New Diagnostics under the auspices of CEPI (*17*). This initiative seeks to build on collaborations with key diagnostic partners to enable adequate provision of diagnostic capabilities during outbreaks. WHO states that IRPs are major resources for ensuring the reliability of in vitro biological diagnostic procedures used for diagnosis of diseases (*18*). The recent epidemics caused by MERS-CoV, Ebola virus, and Zika virus have highlighted complexities in prompt development of sensitive and specific assays, particularly those that would be accessible in resource constrained settings (*19*–*21*). Availability of international reference standards that encompass genetic material, antigens, and antibodies would enable harmonization and calibration of tests. These standards would facilitate development and validation of diagnostic assays, which include nucleic acid amplification tests, serologic assays, and antigen-based point-of-care tests. 

## Development of Vaccines and Therapeutics

No vaccine or therapeutic is currently licensed by regulators in Europe or the United States for use for any disease on the research and development blueprint. On August 8, 2014, WHO declared the Ebola outbreak in West Africa to be a public health emergency of international concern. This declaration led to subsequent concerted efforts from the international scientific community to accelerate development of Ebola vaccines, with funding, regulatory and ethical review, expert advice, and manufacturing support being initiated at extraordinary speed (*22*). Although vaccine development was considered to be a relatively successful component of the Ebola research and development efforts, and the recombinant vesicular stomatitis virus–Zaire Ebola virus showed high efficacy in a ring vaccination trial (*23*), this development came too late to have any major effect on the course of the West Africa epidemic. Other candidate vaccines also underwent clinical evaluation, and encouraging safety and immunogenicity data were generated from phase 1 and 2 trials of several candidates. However, studies were performed by different groups with a variety of immunogenicity assays and reference reagents being used, making comparison of immunogenicity results complicated (*24**–**28*). A set of biological standards that are common to all assays would enable calibration and harmonization of assay data between trials, and could prove vital in determining correlates of protection and clinical outcomes. These standards would also enable better selection of candidate vaccines to transition from the preclinical stage to clinical evaluation.

There has been considerable interest in biological therapies for emerging epidemic threats (*29*). Convalescent-phase plasma and monoclonal antibodies have been evaluated as treatment for Ebola (*30*,*31*) and have been considered as therapies for Zika virus (*32*) and MERS-CoV (*33*,*34*), forming a key focus of the MERS-CoV research and development blueprint roadmap (*12*). Availability of IRPs would enable characterization of the potency, purity, and identity of complex biological materials, such as antibodies, which would facilitate not only research of immunotherapies but also the standardization of preparations postlicensure.

## Challenges in Development of Standards for Research and Development Blueprint Diseases

Antigen and nucleic acid reference materials can be synthesized through recombinant techniques as long as necessary genomic sequence data are available (*35*–*37*), but for some newly emerging pathogens, acquisition of live pathogens might be required. Simple blood draws can complicate field operations in the midst of an epidemic, and logistical issues regarding processing and storage of acute, potentially infectious samples can also present many obstacles. However, the ideal starting material for production of antibody-based reference preparations is serum or plasma from convalescent-phase patients.

Novel methods for generating fully human immunoglobulin for use as reference materials have been explored when human plasma is not readily available, such as inoculation of transchromosomal cattle and subsequent isolation of antibodies (*35*,*38*,*39*). However, this technology is still relatively new, and convalescent-phase serum is considered superior because it will most closely represent a clinical sample and has a polyclonal range of antibody specificities that can be further optimized by pooling samples. Although many previous clinical studies of the research and development blueprint priority diseases have resulted in collection and storage of plasma from convalescent patients, several obstacles would largely preclude these samples being repurposed to generate reference materials. These issues include sample volume, receipt of appropriate consent, appropriate records, documentation of sample provenance, and willingness of researchers to share samples. A more suitable alternative to repurposing old samples would be to initiate clinical studies with objectives that include acquisition of blood samples for production of reference materials for each of the research and development blueprint priority diseases. However, such studies would have several challenges.

## Challenge in Identifying Suitable Patients to Donate Material

Many pathogens on the WHO priority pathogens list cause outbreaks that are difficult to anticipate temporally and geographically, and outbreaks are often limited in numbers of cases and duration. These factors complicate prospective acquisition of appropriate clinical samples for development of reference materials. In addition, these pathogens most commonly cause outbreaks in resource-constrained environments, where poor healthcare infrastructure, limited diagnostic capability, and suboptimal disease reporting systems would limit retrospective identification of suitable patients (*40*). For development of Ebola antibody IRPs, plasma was obtained either from recovered EVD patients in Sierra Leone who were enrolled in a convalescent-phase plasma trial or as donations from countries not directly involved in the outbreak (Italy, Norway, the United Kingdom, and the United States) (*14*,*15*). However, it would be inappropriate to use this example as a model for sample acquisition because it required occurrence of a large and devastating outbreak for a concerted international response to be successful.

Focused efforts to obtain material for the generation of IRPs should be made for high-threat epidemic diseases, and methods to identify and sample patients from previous outbreaks should be explored. In addition, preparation for promptly identifying and sampling patients prospectively given a diagnosis in future outbreaks should be made. This preparation would optimize the likelihood of obtaining high-quality samples for rare and sporadic disease pathogens, such as Nipah virus or Rift Valley fever virus, but would require a systematic and coordinated approach that brings together local investigators with international partners in a transparent and equitable framework. Global initiatives, such as the International Severe Acute Respiratory and Emerging Infections Consortium, exist to facilitate the rapid response to emerging infectious disease and epidemic threats by aiding the sharing of clinical research tools, such as open-access clinical study protocols. Initiatives such as this consortium would be well placed to bring together international partners and coordinate a collaborative framework to outline processes for acquisition of samples for generation of reference materials. Preemptive identification of study sites and preparation of study documents, including template protocols and clinical agreements detailing methods for identifying and recruiting potential donors, sample collection, and processing, and roles and responsibilities would optimize the likelihood of success in this regard.

## Ethics Considerations

In situations in which access to existing samples and appropriate consent for use in reference preparations are unavailable, the process of acquiring new samples for development of reference materials should be registered as a unique study for each prioritized epidemic disease. This process would require ethics approval being sought through appropriate channels, thus enabling appropriate scrutiny by relevant ethics and regulatory bodies in countries coordinating activities in disease-endemic countries where samples are to be collected, and in the countries of international partners. These studies would involve minimal risk to participants because they would not involve administration of an investigational medicinal product and in most instances, only a single venesection would be required.

## Consent

Serum, plasma, and plasma-derived products contain insufficient genetic material to be universally considered as human tissue under legislation. However, they must be considered human biological material. Therefore, informed consent is an indispensable requirement for donations. The donor should receive information concerning all aspects of the study, with emphasis placed on the fact that participation is entirely voluntary. The intended use of the blood components for the generation, storage, and international distribution of reference materials must be explained, in addition to the potential beneficiaries and the procedures involved. Appropriate measures should also be taken to ensure protection of personal data and confidentiality, and these issues must be explained to the donor. Such information should be contained in a detailed participant information sheet that should be provided to the donor in advance of providing consent. Consent processes should consist of all participants signing and dating an informed consent form before any study-specific procedures are performed. A collaborative international framework could include template participant information sheets and informed consent forms that could be readily adapted for disease- and country-specific studies when appropriate.

## Ownership and Equitable Benefit Sharing

Sharing of biological materials is a necessity for rapid research progress, but recent experiences during epidemics have highlighted that concerted efforts to establish acceptable processes for obtaining and sharing reference materials are needed. At the present time, only a small number of institutions manufacture and provide reference materials to WHO for use as IRPs. Therefore, export of samples to these institutions is an essential step in the production of IRPs for epidemic diseases. Initiatives exist to facilitate sharing of benefits arising from the use of, and access to, human and nonhuman genetic material. An example of such an initiative is the Pandemic Influenza Preparedness (PIP) framework launched by WHO in 2011. The PIP framework seeks to address concerns of low- and middle-income countries that sharing of influenza virus specimens with WHO was not matched with assurances that benefits derived from such sharing would be equitably distributed (*41*). Concerns about inequitable sharing of benefits were further exacerbated during the 2009 influenza A(H1N1) pandemic because of unequal access to vaccines (*42*). Despite the example set by the PIP framework, more recent epidemics of other infectious diseases have continued to raise issues with regard to sample sharing. For example, local export restrictions in Brazil prevented sharing of well-characterized samples during the recent Zika virus epidemic, thereby presenting an obstacle to rapid development and assessment of diagnostics (*43*,*44*).

Further generalized guidance on fair and equitable sharing of benefits arising out of use of genetic biological resources is in the Nagoya Protocol (*45*). This protocol is a supplementary agreement to the Convention on Biological Diversity and contains detailed guidance divided into the following objectives: access obligations, benefit sharing obligations, and compliance obligations. No similar guidance exists for plasma-derived products, such as antibody reference materials. However, many facets of the Nagoya Protocol would remain pertinent to plasma-derived products and could be adapted to inform procedures.

Acceptance that human-derived products should not be an object of commercialization or source of profit is enshrined in several key documents (*46*,*47*) that would continue to apply to development of IRPs. WHO distributes IRPs either free of charge to National Control Laboratories or with small handling charges and shipping costs to other organizations. Negotiations between relevant parties and preemptive interaction between researchers and donor country authorities could lead to clearly defined, mutually agreed upon processes for acquisition and export of samples. These processes could subsequently be specified in legally binding clinical study agreements, and thus ensure compliance with principles of equitable benefit sharing and provide solutions to complex issues, such as intellectual property, product ownership, and access rights to IRPs.

## Operational Considerations

Most diseases on the research and development blueprint priority disease list are caused by category A pathogens, and handling of potentially infectious materials often requires Biosafety Level 4 laboratories. In recent years, the threat of bioterrorism has resulted in strict rules and requirements, such as US Federal Select Agent regulations, which can result in increased cost, limited research, and reduced collaboration between institutions (*48*). However, there is an encouraging increase in the number of Biosafety Level 4 laboratories globally, and initiatives now exist that seek to harmonize practices and facilitate collaboration between laboratories with the ultimate goal of positively contributing to global health (*49*,*50*). The safe acquisition, processing, exporting, and importing of samples from recovered emerging high-threat disease patients would require careful planning. Requirements would be based on natural history of a specific disease, study site, and patient characteristics, such as period of convalescence since the acute illness. Liaison with national authorities in donor and recipient countries with regard to exportation and importation regulations and requirements would be necessary. In addition, most of these diseases occur primarily in low- and middle-income countries and often in remote or rural settings, where access to accurate medical records and diagnostic tools will be limited. Clear specifications regarding method of diagnosis, patient clinical details and disease course, sample collection date, and storage details would be required.

## Conclusions

Biological IRPs for infectious diseases with epidemic potential are a major global asset that will support timely and efficient development of vaccines, therapeutics, and diagnostics. A critical barrier to development of reference materials for epidemic infections is acquisition of suitable source material. We have highlighted a series of key issues that need to be addressed systematically in a framework that is acceptable to all parties. Work should begin to develop and agree to such a framework and to generate IRPs for these diseases so that drugs, diagnostics, and vaccines are available for future outbreaks.

## References

[1] Page M, Wilkinson DE, Mattiuzzo G, Efstathiou S, Minor P. Developing biological standards for vaccine evaluation. Future Virol. 2017;12:431–7. 10.2217/fvl-2017-0003

[2] World Health Organization. Recommendations for the preparation, characterization and establishment of intenational and other biological reference standards. Geneva: The Organization; 2006.

[3] Ehrlich and Von Behring. Ehrlich and Von Behring. JAMA. 2017;317:1381. 10.1001/jama.2017.0642 28384815

[4] Roux at the Institut Pasteur. The antitoxin treatment of diphtheria: M. BMJ. 1894;2:931–3. 10.1136/bmj.2.1765.931 20755144 PMC2405444

[5] Bosch F, Rosich L. The contributions of Paul Ehrlich to pharmacology: a tribute on the occasion of the centenary of his Nobel Prize. Pharmacology. 2008;82:171–9. 10.1159/000149583 18679046 PMC2790789

[6] Bangham DRHH. H.H. Dale and the London Centre for WHO standards—some milestones of the early years. Dev Biol Stand. 1999;100:11–5. 10616171

[7] Cockburn WC. The international contribution to the standardization of biological substances. I. Biological standards and the League of Nations 1921-1946. Biologicals. 1991;19:161–9. 10.1016/1045-1056(91)90030-N 1953999

[8] Kieny MP, Rottingen JA, Farrar J; WHO R&D Blueprint team; R&D Blueprint Scientific Advisory Group. The need for global R&D coordination for infectious diseases with epidemic potential. Lancet. 2016;388:460–1. 10.1016/S0140-6736(16)31152-7 27507751 PMC7133575

[9] World Health Organization. A research and development blueprint for action to prevent epidemics [cited 2018 Nov 7]. https://www.who.int/blueprint/en

[10] World Health Organization. Annual review of diseases prioritized under the research and development blueprint: informal consultation., 2017 [cited 2018 Nov 7]. https://www.who.int/emergencies/diseases/2018prioritization-report.pdf

[11] World Health Organization. List of blueprint priority diseases; 2018 [cited 2018 Nov 7]. https://www.who.int/blueprint/priority-diseases/en

[12] Modjarrad K, Moorthy VS, Ben Embarek P, Van Kerkhove M, Kim J, Kieny MP. A roadmap for MERS-CoV research and product development: report from a World Health Organization consultation. Nat Med. 2016;22:701–5. 10.1038/nm.4131 27387881 PMC7096003

[13] Plotkin SA. Vaccines for epidemic infections and the role of CEPI. Hum Vaccin Immunother. 2017;13:2755–62. 10.1080/21645515.2017.1306615 28375764 PMC5718831

[14] World Health Organization. WHO collaborative study to assess the suitability of an interim standard for antibodies to Ebola virus, 2015 [cited 2018 Nov 7]. http://apps.who.int/iris/handle/10665/197777

[15] Wilkinson DE, Hassall M, Mattiuzzo G, Stone L, Atkinson E, Hockley J, et al. WHO collaborative study to assess the suitability of the first international standard and the first international reference panel for antibodies to Ebola virus, 2017 [cited 2018 Nov 7]. https://www.who.int/biologicals/expert_committee/BS2316_Anti-EBOV_Antibodies_WHO_1st_IS_and_WHO_1st_International_Ref_Panel.pdf

[16] Li C, Xu K, Hashem A, Shao M, Liu S, Zou Y, et al. Collaborative studies on the development of national reference standards for potency determination of H7N9 influenza vaccine. Hum Vaccin Immunother. 2015;11:1351–6. 10.1080/21645515.2015.1032490 25970793 PMC4514420

[17] Perkins MD, Dye C, Balasegaram M, Bréchot C, Mombouli JV, Røttingen JA, et al. Diagnostic preparedness for infectious disease outbreaks. Lancet. 2017;390:2211–4. 10.1016/S0140-6736(17)31224-2 28577861

[18] World Health Organization. International reference materials [cited 2018 Nov 7]. https://www.who.int/bloodproducts/ref_materials/en

[19] Chan JF, Sridhar S, Yip CC, Lau SK, Woo PC. The role of laboratory diagnostics in emerging viral infections: the example of the Middle East respiratory syndrome epidemic. J Microbiol. 2017;55:172–82. 10.1007/s12275-017-7026-y 28243939 PMC7090747

[20] Shorten RJ, Brown CS, Jacobs M, Rattenbury S, Simpson AJ, Mepham S. Diagnostics in Ebola virus disease in resource-rich and resource-limited settings. PLoS Negl Trop Dis. 2016;10:e0004948. 10.1371/journal.pntd.0004948 27788135 PMC5082928

[21] Waggoner JJ, Pinsky BA. Zika virus: diagnostics for an emerging pandemic threat. J Clin Microbiol. 2016;54:860–7. 10.1128/JCM.00279-16 26888897 PMC4809954

[22] Venkatraman N, Silman D, Folegatti PM, Hill AVS. Vaccines against Ebola virus. Vaccine. 2017. 28780120 10.1016/j.vaccine.2017.07.054

[23] Henao-Restrepo AM, Camacho A, Longini IM, Watson CH, Edmunds WJ, Egger M, et al. Efficacy and effectiveness of an rVSV-vectored vaccine in preventing Ebola virus disease: final results from the Guinea ring vaccination, open-label, cluster-randomised trial (Ebola Ça Suffit!). Lancet. 2017;389:505–18. 10.1016/S0140-6736(16)32621-6 28017403 PMC5364328

[24] Ewer K, Rampling T, Venkatraman N, Bowyer G, Wright D, Lambe T, et al. A monovalent chimpanzee adenovirus Ebola vaccine boosted with MVA. N Engl J Med. 2016;374:1635–46. 10.1056/NEJMoa1411627 25629663 PMC5798586

[25] Tapia MD, Sow SO, Lyke KE, Haidara FC, Diallo F, Doumbia M, et al. Use of ChAd3-EBO-Z Ebola virus vaccine in Malian and US adults, and boosting of Malian adults with MVA-BN-Filo: a phase 1, single-blind, randomised trial, a phase 1b, open-label and double-blind, dose-escalation trial, and a nested, randomised, double-blind, placebo-controlled trial. Lancet Infect Dis. 2015. 26546548 10.1016/S1473-3099(15)00362-X PMC4700389

[26] Milligan ID, Gibani MM, Sewell R, Clutterbuck EA, Campbell D, Plested E, et al. Safety and immunogenicity of novel adenovirus type 26- and modified vaccinia Ankara-vectored Ebola vaccines: a randomized clinical trial. JAMA. 2016;315:1610–23. 10.1001/jama.2016.4218 27092831

[27] Regules JA, Beigel JH, Paolino KM, Voell J, Castellano AR, Hu Z, et al.; rVSVΔG-ZEBOV-GP Study Group. rVSVΔG-ZEBOV-GP Study Group. A recombinant vesicular stomatitis virus Ebola vaccine. N Engl J Med. 2017;376:330–41. 10.1056/NEJMoa1414216 25830322 PMC5408576

[28] Zhu FC, Wurie AH, Hou LH, Liang Q, Li YH, Russell JB, et al. Safety and immunogenicity of a recombinant adenovirus type-5 vector-based Ebola vaccine in healthy adults in Sierra Leone: a single-centre, randomised, double-blind, placebo-controlled, phase 2 trial. Lancet. 2017;389:621–8. 10.1016/S0140-6736(16)32617-4 28017399

[29] Moekotte AL, Huson MA, van der Ende AJ, Agnandji ST, Huizenga E, Goorhuis A, et al. Monoclonal antibodies for the treatment of Ebola virus disease. Expert Opin Investig Drugs. 2016;25:1325–35. 10.1080/13543784.2016.1240785 27676206

[30] van Griensven J, Edwards T, de Lamballerie X, Semple MG, Gallian P, Baize S, et al.; Ebola-Tx Consortium. Ebola-Tx Consortium. Evaluation of convalescent plasma for Ebola virus disease in Guinea. N Engl J Med. 2016;374:33–42. 10.1056/NEJMoa1511812 26735992 PMC5856332

[31] Davey RT Jr, Dodd L, Proschan MA, Neaton J, Neuhaus Nordwall J, Koopmeiners JS, et al.; Multi-National PREVAIL II Study Team. Multi-National PREVAIL II Study Team. A randomized, controlled trial of ZMapp for Ebola virus infection. N Engl J Med. 2016;375:1448–56. 10.1056/NEJMoa1604330 27732819 PMC5086427

[32] Munjal A, Khandia R, Dhama K, Sachan S, Karthik K, Tiwari R, et al. Advances in developing therapies to combat Zika virus: current knowledge and future perspectives. Front Microbiol. 2017;8:1469. 10.3389/fmicb.2017.01469 28824594 PMC5541032

[33] Arabi YM, Hajeer AH, Luke T, Raviprakash K, Balkhy H, Johani S, et al. Feasibility of using convalescent plasma immunotherapy for MERS-CoV infection, Saudi Arabia. Emerg Infect Dis. 2016;22:1554–61. 10.3201/eid2209.151164 27532807 PMC4994343

[34] Mustafa S, Balkhy H, Gabere MN. Current treatment options and the role of peptides as potential therapeutic components for Middle East Respiratory Syndrome (MERS): a review. J Infect Public Health. 2017. 28864360 10.1016/j.jiph.2017.08.009 PMC7102797

[35] Felder E, Wölfel R. Development of a versatile and stable internal control system for RT-qPCR assays. J Virol Methods. 2014;208:33–40. 10.1016/j.jviromet.2014.07.028 25072380

[36] Mattiuzzo G, Ashall J, Doris KS, MacLellan-Gibson K, Nicolson C, Wilkinson DE, et al. Development of lentivirus-based reference materials for Ebola virus nucleic acid amplification technology-based assays. PLoS One. 2015;10:e0142751. 10.1371/journal.pone.0142751 26562415 PMC4642882

[37] Pasloske BL, Walkerpeach CR, Obermoeller RD, Winkler M, DuBois DB. Armored RNA technology for production of ribonuclease-resistant viral RNA controls and standards. J Clin Microbiol. 1998;36:3590–4. 9817878 10.1128/jcm.36.12.3590-3594.1998 PMC105245

[38] Wilkinson DE, Page M, Mattiuzzo G, Hassall M, Dougall T, Rigsby P, et al. Comparison of platform technologies for assaying antibody to Ebola virus. Vaccine. 2017;35:1347–52. 10.1016/j.vaccine.2016.11.083 28161420 PMC5322821

[39] Dye JM, Wu H, Hooper JW, Khurana S, Kuehne AI, Coyle EM, et al. Production of potent fully human polyclonal antibodies against Ebola Zaire virus in transchromosomal cattle. Sci Rep. 2016;6:24897. 10.1038/srep24897 27109916 PMC4842964

[40] Gostin LO, Tomori O, Wibulpolprasert S, Jha AK, Frenk J, Moon S, et al. Toward a common secure future: four global commissions in the wake of Ebola. PLoS Med. 2016;13:e1002042. 10.1371/journal.pmed.1002042 27195954 PMC4873000

[41] Fidler DP, Gostin LO. The WHO pandemic influenza preparedness framework: a milestone in global governance for health. JAMA. 2011;306:200–1. 10.1001/jama.2011.960 21750298

[42] Fidler DP. Negotiating equitable access to influenza vaccines: global health diplomacy and the controversies surrounding avian influenza H5N1 and pandemic influenza H1N1. PLoS Med. 2010;7:e1000247. 10.1371/journal.pmed.1000247 20454566 PMC2864298

[43] Haug CJ, Kieny MP, Murgue B. The Zika challenge. N Engl J Med. 2016;374:1801–3. 10.1056/NEJMp1603734 27028782

[44] Cheng M, Satter R, Goodman J. Health officials want more Zika samples, data from Brazil. The Associated Press, 2016 [cited 2018 Nov 7]. https://www.businessinsider.com/ap-health-officials-want-more-zika-samples-data-from-brazil-2016-2

[45] Diversity SOTCOB. Nagoya protocol on access to genetic resources and the fair and equitable sharing of benefits arising from their utilization to the convention on biological diversity [cited 2018 Nov 7]. https://www.cbd.int/abs/doc/protocol/nagoya-protocol-en.pdf

[46] Council of Europe. Convention for the protection of human rights and dignity of the human bing with regard to the application of biology and medicine: convention on human rights and biomedicine. Treaty no. 1641997 [cited 2018 Nov 7]. https://rm.coe.int/168007cf98

[47] Final report to the European Commission on the project Basic Ethical Principles in Bioethics and Biolaw, 1995–1998 Part B 1999 [cited 2018 Nov 7]. http://cometc.unibuc.ro/reglementari/Basic-Ethical-Principles.pdf

[48] Wurtz N, Grobusch MP, Raoult D. Negative impact of laws regarding biosecurity and bioterrorism on real diseases. Clin Microbiol Infect. 2014;20:507–15. 10.1111/1469-0691.12709 24909400

[49] Nisii C, Castilletti C, Raoul H, Hewson R, Brown D, Gopal R, et al. Biosafety Level-4 laboratories in Europe: opportunities for public health, diagnostics, and research. PLoS Pathog. 2013;9:e1003105. 10.1371/journal.ppat.1003105 23349630 PMC3547859

[50] Reusken CB, Mögling R, Smit PW, Grunow R, Ippolito G, Di Caro A, et al. Status, quality and specific needs of Ebola virus diagnostic capacity and capability in laboratories of the two European preparedness laboratory networks EMERGE and EVD-LabNet. Euro Surveill. 2018;23:23. 10.2807/1560-7917.ES.2018.23.19.17-00404 29766839 PMC5954606

